# Exposure to PCB126 during the nursing period reversibly impacts early-life glucose tolerance

**DOI:** 10.3389/fendo.2023.1085958

**Published:** 2023-03-22

**Authors:** Brittany B. Rice, Keegan W. Sammons, Sara Y. Ngo Tenlep, Madeline T. Weltzer, Leryn J. Reynolds, Cetewayo S. Rashid, Hollie I. Swanson, Kevin J. Pearson

**Affiliations:** ^1^ Department of Pharmacology and Nutritional Sciences, University of Kentucky, Lexington, KY, United States; ^2^ Human Movement Sciences, Darden College of Education, Old Dominion University, Norfolk, VA, United States

**Keywords:** obesity, polychlorinated biophenyls, diabetes, mice, developmental programming of adult disease, DOHaD (development origins of health and disease), lactation

## Abstract

Polychlorinated biphenyls (PCBs) are persistent environmental organic pollutants known to have detrimental health effects. Using a mouse model, we previously demonstrated that PCB126 exposure before and during pregnancy and throughout the perinatal period adversely affected offspring glucose tolerance and/or body composition profiles. The purpose of this study was to investigate the glucose tolerance and body composition of offspring born to dams exposed to PCB126 during the nursing period only. Female ICR mice were bred, and half of the dams were exposed to either vehicle (safflower oil) or 1 µmole PCB126 per kg of body weight *via* oral gavage on postnatal days (PND) 3, 10, and 17 (n = 9 per group). Offspring body weight, lean and fat mass, and glucose tolerance were recorded every three weeks. PCB126 treatment did not alter dam nor offspring body weight (p > 0.05). PCB126-exposed male and female offspring displayed normal body composition (p > 0.05) relative to vehicle-exposed offspring. However, both male and female offspring that were exposed to PCB126 during the nursing period had significantly impaired glucose tolerance at 3 and 9 weeks of age (p < 0.05). At 6 and 12 weeks of age, no impairments in glucose tolerance existed in offspring (p > 0.05). Our current study demonstrates that exposure to PCB126 through the mother’s milk does not affect short- or long-term body composition but impairs glucose tolerance in the short-term.

## 1 Introduction

Polychlorinated biphenyls (PCBs) are a class of chlorinated hydrocarbons that were once widely manufactured for use in numerous industrial and commercial products because of their chemical stability, heat resistance, and electrical insulating properties (1). Upon accumulating evidence demonstrating the detrimental effects PCB exposure had on health, the United States banned the production of PCBs in the late 1970s (2). Despite cessation in production, PCBs continue to pose a threat to human health due to their persistent environmental presence (3). PCBs are ubiquitous and are found in almost every medium within the environment despite the fact that there are no known natural sources of the toxic compounds (4–7). Routes of PCB exposure include dermal contact, inhalation, and ingestion (3). The environmental fates of PCBs are largely determined by their chlorination pattern (6). Over 200 congeners exist and differ by their number and placement of chlorine groups on the phenyl rings. The number of chlorine group attachments and their substitutions influence the toxicity and persistence of the PCB mixtures (8). Non-ortho substituted PCBs or coplanar PCBs are more resistant to biodegradation and biotransformation (6). Thus, the introduction of such PCBs to our ecosystems through environmental uptake results in biomagnification as PCBs accumulate in adipose tissue (6, 8).

Coplanar PCBs are known as ‘dioxin-like’ PCBs because they activate the aryl hydrocarbon receptor (AhR) (9), the receptor responsible for initiating the biotransformation of many polyhalogenated xenobiotics. PCB126 (3, 3’, 4, 4’, 5-pentachlorobiphenyl) is the most potent AhR agonist among the PCB family (10). Observed epidemiologic associations between PCB exposure and diabetes prevalence (11–13), incidence (14–16), and risk (17–19) have been corroborated by laboratory investigations. Specifically, coplanar PCB exposure has been demonstrated to promote obesity (20–22), increase insulin resistance (21, 22), and impair glucose tolerance (23–26).

Further, PCBs can transfer from mother to child during the perinatal period by crossing the placenta as well as entering breastmilk (27). Because of this, understanding how early-life PCB exposure affects the developing fetus and infant is of paramount significance. The Developmental Origins of Health and Disease hypothesis, derived from the Barker hypothesis, postulates that early-life stressors influence health outcomes observed in later life (28). Research documents that the programming of disease can occur before birth and in the early postnatal period (29). However, literature scantly details the contribution of early-life PCB exposures to diabetic-like phenotypes observed in offspring. Specifically, offspring exposed to PCB126 during gestation and nursing exhibit sex-specific alterations in body composition (30). While likely acting through different mechanisms than PCB126, PCB153 exposure during gestation and lactation caused sex-specific alterations in metabolic parameters in offspring (31). PCB126 exposure during gestation results in long-lasting alterations in offspring body weight, body composition, and glucose tolerance (32). Investigation of embryonic and fetal PCB126 exposures demonstrates the ability of the toxicant to modify beta cell development (33) and the profiles of pro-inflammatory cytokines and hormones implicated in glucose regulation (34). In the current study, we exposed dams to PCB126 during the nursing period in an effort to delineate how nursing PCB126 exposures influence body composition and glucose homeostasis in offspring. Based on our previous studies of perinatal PCB126 exposure (30), we hypothesized that PCB exposure *via* nursing during early postnatal life will unfavorably alter offspring body composition and therefore impair offspring glucose tolerance.

### 1.1 Animals, breeding, and PCB treatment

Ten-week-old male and female ICR mice (Envigo, Indianapolis, IN) were housed in a pathogen-free environment in NextGen 500 cages (Allentown Inc., Allentown, NJ) under a 14:10 light/dark cycle, with temperatures ranging from 20 - 22.2˚ Celsius, and humidity ranging from 30% to 70%. The ICR stock was selected based on its exceptional reproductive abilities, extensive use in toxicological and pharmacological studies, and our previous characterizations of maternal PCB126 exposure effects on offspring glucose tolerance and body composition (30, 32). Female mice were singly housed for six days prior to mating. Each female mouse was exposed to male bedding (Sani-Chip Bedding, Teklad 7115, Envigo, Indianapolis, IN) for two days before being allowed to mate. One male mouse was placed in a cage with two female mice for 24 hours. Dams were allowed to spontaneously deliver. Litters were culled to four pups (two pups per sex) on postnatal day (PND) 1.

On PNDs 3, 10, and 17, dams were administered either tocopherol stripped safflower oil (vehicle) (Dyets #403952, Bethlehem, PA), or one micromole of PCB126 per kilogram body weight (AccuStandard Inc., New Haven, CT) dissolved in vehicle *via* oral gavage (n = 9 mice per treatment group). As is typical of developmental programming studies, offspring sample size indicates the number of dams/litters. Dams were selected and assigned to experimental groups according to the date of birth in order to match vehicle and PCB treatment groups as evenly as possible. Offspring exposure to vehicle or PCB126 occurred through milk during nursing. Offspring were microchipped for identification purposes (IPTT-300 Implantable Programmable Temperature Transponder, BDMS, Seaford, DE). Offspring were weaned on PND 21 and group housed with same sex pups from other dams of the same treatment group. Throughout the duration of the study, body weights were monitored weekly, and mice were fed standard chow (Teklad 2918 Global Rodent Diet, Envigo, Madison, WI) and had *ad libitum* access to food and water. Body weight data are provided for those weeks when the glucose tolerance testing and EchoMRI were performed. Offspring were euthanized at 42-44 weeks of age. All animal procedures performed during this study were approved by the University of Kentucky Institutional Animal Care and Use Committee.

### 1.2 Glucose tolerance testing

Every three weeks, beginning on PND 21, glucose tolerance tests were performed on offspring. Prior to testing, animals were fasted for three hours and had their tail vein pricked. An intraperitoneal injection of two grams per kilogram body weight of dextrose (Bimeda Inc., Le Sueur, MN) was given to one mouse of each sex per litter. Prior to the injection (0 minutes) and 15, 30, 60, and 120 minutes post-injection, a hand-held glucometer (Bayer Breeze 2, Bayer Health Care LLC, Tarrytown, NY) was used to quantify blood glucose levels. The total area under the curve (AUC) for blood glucose levels were calculated using the ‘Area Below Curves’ function in SigmaPlot 14.0.

### 1.3 Body composition analyses

Total fat mass, lean mass, and water were measured in live, conscious male and female offspring by nuclear magnetic resonance (EchoMRI; Echo Medical Systems, Houston, TX) every three weeks.

### 1.4 Statistics

**Figure 1:**
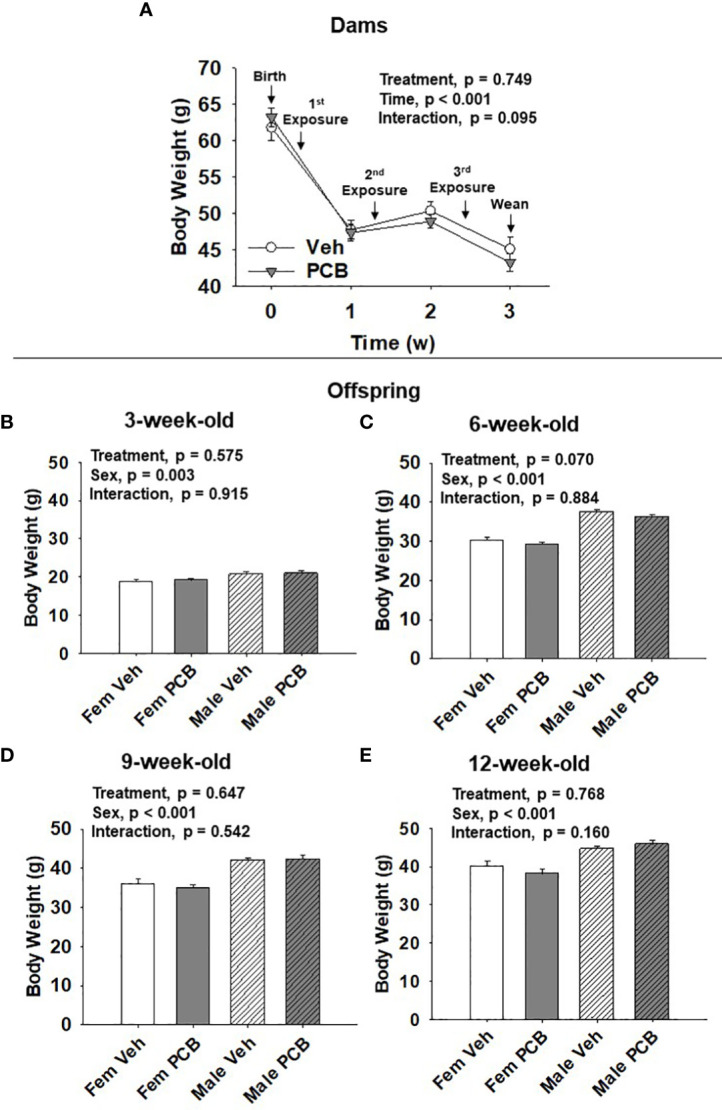
PCB126 exposure during the nursing period does not affect dam or offspring body weight. **(A)** The body weight of vehicle and PCB126-treated dams from parturition to weaning are shown. Dams received treatments respective to their experimental grouping 3, 10, and 17 days after delivery. Dam body weight values are representative of the mean ± SEM (n = 9 mice per group). The body weight of **(B)** 3-week-old **(C)** 6-week-old, **(D)** 9-week-old, and **(E)** 12-week-old offspring born to dams exposed to PCB126 or vehicle during the nursing period are shown. Offspring body weight values are provided as means of litter means ± SEM (n = 9 per group). Two-factor repeated measures ANOVA and two-factor ANOVA were respectively used to analyze dam and offspring body weight.

**Figure 2:**
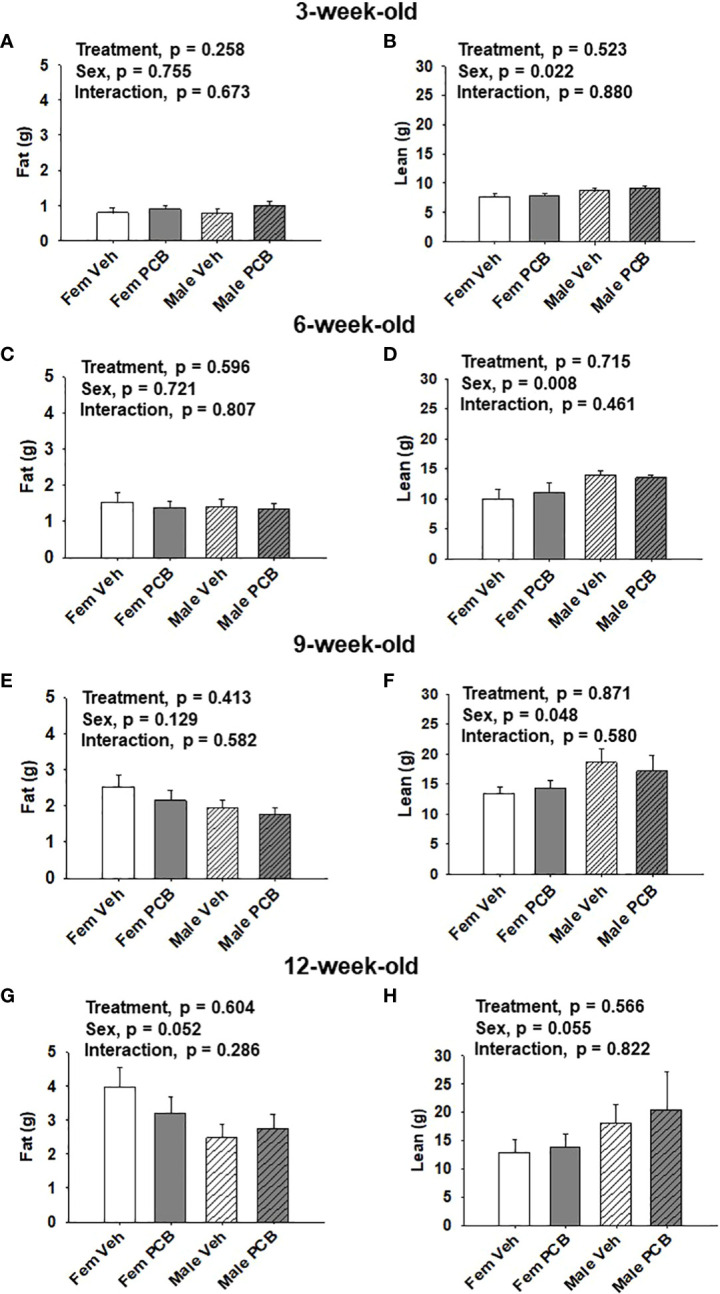
Maternal PCB126 exposure during the nursing period does not alter body composition. Body composition (lean and fat mass) of offspring was measured at 3, 6, 9, and 12 weeks of age *via* EchoMRI. Shown are the offspring data from 3 **(A, B)**, 6 **(C, D)**, 9 **(E, F)** and 12 **(G, H)** weeks of age. Fat mass and lean mass values are presented as the mean of litter means ± SEM (n = 9 per group). Data shown were analyzed using two-factor ANOVA.

**Figure 3:**
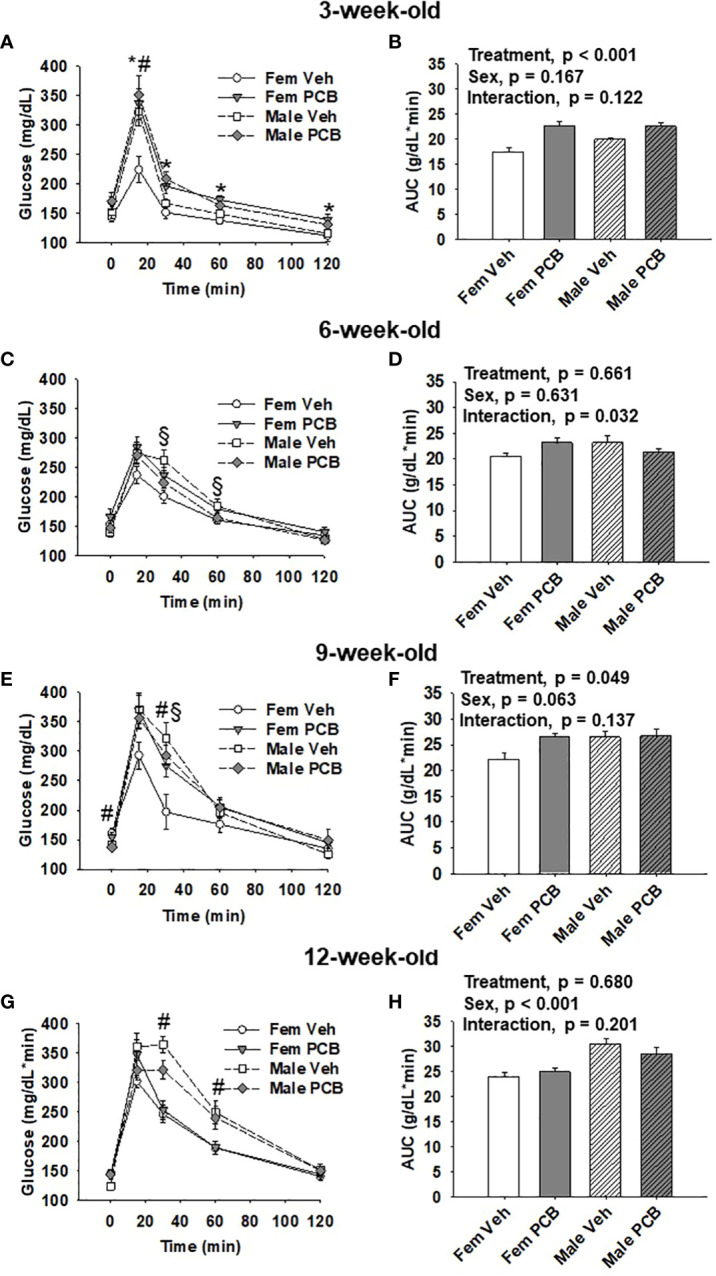
Offspring born to PCB126-exposed dams exhibit short-term impairments in glucose tolerance. Measurements of glucose levels in offspring are shown in response to intraperitoneal glucose injection. The glucose tolerance testing of **(A)** 3-week-old, **(C)** 6-week-old, **(E)** 9-week-old, and **(G)** 12-week-old vehicle- and PCB-exposed offspring. Significant sex-specific differences and treatment differences in glucose tolerance were respectively observed in male offspring and PCB126-exposed offspring (**A**, **E**, and **G**; p < 0.05 for all). Total AUC of **(B)** 3-week-old and **(D)** 6-week-old, **(F)** 9-week-old, and **(H)** 12-week-old vehicle- and PCB-exposed offspring blood glucose levels are shown. Significant differences in treatment were observed in offspring at 3 weeks of age (**B**, p < 0.001) and 9 weeks of age (**D**, p = 0.049), while significant differences in sex was observed at 12 weeks of age (**H**, p < 0.001). Values are shown as the mean ± SEM (n = 9 per group). Two-factor ANOVA was used to analyze glucose tolerance and AUC data. Specifically, two-factor ANOVA was used at each time-point for glucose tolerance data analyses. The # sign designates a main effect of sex while the * designates a main effect of PCB treatment (P < 0.05). Significant interactions between treatment and sex are indicated by §.

All analyses were completed using SigmaPlot 14.0. Dam body weight was analyzed using two-factor repeated measures ANOVA and offspring body weight, body composition, glucose tolerance testing, and AUC data were analyzed using two-factor ANOVA, where the factors of analysis were treatment and time or sex. Fisher’s Least Significance Difference *post-hoc* testing was employed upon the detection of interactions between the factors. Significance was set at α < 0.05 for all comparisons. When parameters set for normality were not met (p < 0.05), data were natural log or square root transformed. Upon data transformations, dams body weight data (depicted in **Figure 1A**), offspring 6-week-old lean mass data (depicted in **Figure 2D**), and offspring 9-week-old lean mass data (depicted in **Figure 2F**) still failed to obtain equal variances between samples. Certain data failed to obtain a normal distribution following transformation: offspring 9-week-old body weight (depicted in **Figure 1D**), 12-week-old offspring lean mass data (depicted in **Figure 2H**), offspring 6-week-old 0 min and 9-week-old 120 min glucose tolerance (respectively depicted in **Figures 3C, E**). As a result, the probability (p) values reported in **Figures 1A, D**, **2D, F, H**, **3C** at 0 min, and **Figure 3E** at 120 min were obtained using untransformed data.

## 2 Results

### 2.1 Dam and offspring body weight

Dam body weight changed significantly over time (**Figure 1A**, p < 0.001) respective to weight loss after delivery and while nursing. PCB treatment was found to not significantly affect dam body weight (**Figure 1A**, p = 0.749). No interaction between treatment and time in dam body weight was observed (**Figure 1A**, p = 0.095). Offspring body weights were measured from 3 to 12 weeks of age. Body weight gain was apparent as offspring aged (**Figures 1B–E**). Male offspring weighed significantly more than female offspring throughout the study (**Figure 1B**, p = 0.003; **Figure 1C**, p < 0.001; **Figure 1D**, p < 0.001; and **Figure 1E**, p < 0.001). Nursing exposure to PCB126 did not result in any difference in the body weight of offspring during the study (**Figure 1B**, p = 0.575; **Figure 1C**, p = 0.070; **Figure 1D**, p = 0.647; and **Figure 1E**, p = 0.768). No interactions between sex and treatment were observed in regards to offspring body weight (**Figure 1B**, p = 0.915; **Figure 1C**, p = 0.884; **Figure 1D**, p = 0.542; and **Figure 1E**, p = 0.160).

### 2.2 Offspring body composition

No differences in offspring fat or lean mass respective to PCB treatment were observed throughout the study (**Figure 2A**, p = 0.258; **Figure 2B**, p = 0.523; **Figure 2C**, p = 0.596; **Figure 2D**, p = 0.715; **Figure 2E**, p = 0.413; **Figure 2F**, p = 0.871; **Figure 2G**, p = 0.604; and **Figure 2H**, p = 0.566). However, sex-specific differences in lean mass amount were present at 3, 6, and 9 weeks of age, where female offspring had lower lean mass compared to male offspring (**Figure 2B**, p = 0.022; **Figure 2D**, p = 0.008; and **Figure 2F**, p = 0.048). At 12 weeks of age, marginal sex effects were observed where female offspring exhibit a trend towards increased fat mass compared to male offspring (**Figure 2G**, p = 0.052) and male offspring exhibit a trend towards higher lean mass compared to female offspring (**Figure 2H**, p = 0.055). No interactions between sex and treatment were detected regarding offspring lean and fat mass profiles (**Figure 2A**, p = 0.673; **Figure 2B**, p = 0.880; **Figure 2C**, p = 0.807; **Figure 2D**, p = 0.461; **Figure 2E**, p = 0.582; **Figure 2F**, p = 0.580; **Figure 2G**, p = 0.286; and **Figure 2H**, p = 0.822).

### 2.3 Offspring glucose tolerance

At 3 weeks of age, postnatal PCB exposure caused significant impairments in glucose disposal at all time-points during testing beyond fasting in both female and male offspring compared to vehicle control offspring (**Figure 3A**, p < 0.05). At 15 minutes following the glucose challenge, male offspring exhibited significantly elevated blood glucose levels when compared to female offspring (**Figure 3A**, p < 0.05). Glucose disposal curves generated from glucose tolerance testing were summarized using AUC, and both male and female PCB-exposed offspring had higher AUC values than those vehicle-exposed offspring (**Figure 3B**, p < 0.001). Additionally, no sex differences or interactions were detected in the AUC data (**Figure 3B**, p > 0.100). At six weeks of age, no differences in glucose disposal of offspring respective to treatment or sex were observed (**Figure 3C**, p > 0.05). Cross-over interactions between sex and treatment were detected at 30 minutes and 60 minutes during testing (**Figure 3C**, p = 0.009 and p = 0.027 respectively). Likewise, AUC values for offspring did not differ based on treatment or sex (**Figure 3D**, p > 0.600), but a significant interaction between treatment and sex was detected (**Figure 3D**, p = 0.032). At 9 weeks of age, treatment did not influence glucose disposal profiles at individual time-points during testing (**Figure 3E**, p > 0.05). An interaction between treatment and sex was detected at the 30-minute time-point (**Figure 3E**, p = 0.030), where female offspring exposed to PCB exhibited pronounced elevations in blood glucose levels when compared to vehicle-exposed female offspring (p = 0.025). While AUC data did not reveal any significant differences in offspring AUC values based on sex (**Figure 3F**, p = 0.063), PCB-exposed offspring had higher AUC values when compared to vehicle controls (**Figure 3F**, p = 0.049). No interactions between sex and treatment were detected (**Figure 3F**, p = 0.137). Glucose tolerance testing at 12 weeks of age yielded no significant differences between groups respective to treatment (**Figure 3G**, p > 0.1). Offspring AUC data at 12 weeks of age demonstrate that glucose disposal differed by sex (**Figure 3H**, p < 0.001), not treatment (**Figure 3H**, p = 0.680). No interactions between treatment and sex were detected (**Figure 3H**, p = 0.201).

## 3 Discussion

Type 2 diabetes (T2D), a disease in which affected individuals have elevated blood glucose levels due to insulin impairments, is one of the leading causes of death globally (35). Progressive elevations in the prevalence of T2D within child and adolescent populations (36) have gathered the attention of many to the etiology of the disease. Risk factors for T2D have extended beyond hallmarked contributions of genetics and lifestyle and now include environmental exposures (37). Despite piqued interest, research has failed to sufficiently detail the underlying mechanisms by which environmental exposure to PCBs contribute to the developmental programming of T2D. The current study used glucose tolerance testing to assess diabetes risk in offspring in relation to PCB126 exposure during nursing (early postnatal life). Results from the present study demonstrate that offspring exposed to PCB126 during the nursing period exhibited short-term impairments in early-life glucose tolerance despite displaying normal body composition and body weight gain while aging to 12 weeks. This finding extends the current body of etiological evidence supporting the contribution of early-life PCB exposure to the development of diabetic-like phenotypes (30–34), as well as provides insight into understanding how the timing of PCB exposure during development affects diabetic-like outcomes observed later in life.

Previously, we conducted dosing studies to understand the effects of perinatal PCB exposure on offspring diabetes and obesity health outcomes in which ICR dams were exposed to 0, 0.5, or 1 micromole of PCB126 per kilogram body weight *via* oral gavage at a frequency of once per every 14 days beginning two days before mating. There were a total of three exposures: once prior to mating, during gestation, and during nursing (30), and our goal in the current study was to replicate the number of exposures but during the nursing period only. Despite differences in exposure paradigms between our previous work and the current study, our current observation of comparable body weights between animals exposed to vehicle and PCB126 during the nursing period are corroborated by our finding from our preceding study when dams were exposed to PCB126 throughout the perinatal period (30). Mennigen et al. (38) found the pre-weaning and adolescent body weights of Sprague-Dawley rats exposed during gestation days 16 and 18 to Aroclor 1221, a commercial PCB mixture, were not significantly different from that of the control animals. Offspring gestational PCB mixture exposure occurred through placental transfer of dams exposed intraperitoneally to 1 milligram/kilogram of Aroclor 1221 (38). Interestingly, in the same study, the body weights of offspring of the F2 and F3 generations were elevated as a result of the F0 PCB gestational exposure (38). In C57BL/6 J mouse dams exposed to 3.0 micrograms/kilogram body weight of dioxin *via* oral gavage during gestation day 12.5 of pregnancy, toxicant exposure was not shown to affect the body weight of exposed offspring as they aged (39). This lack of effect may be due to a single exposure. Independent of perinatal exposures, PCBs have been demonstrated to increase body weight (20, 22, 40–42). Studies demonstrating the impact of PCB exposure to body composition indicate that PCB-induced obesity may occur *via* alteration of adipocyte development (20, 21, 43, 44) and death (44). However, the route, dose, and timing of coplanar/non-coplanar PCBs or mixtures exposures must be considered to avoid overinterpretation of the comparisons to previous studies.

Although excess body weight has been linked to diabetes development and progression, body composition analyses provide a more comprehensive understanding of diabetes risk and glycemic control. Individuals possessing increased fat mass and reduced lean mass may be more susceptible to hyperglycemia. In adult male mice, direct PCB exposure has been demonstrated to increase fat mass (42). Here, we report no differences in the early-life body composition of offspring exposed to PCB126 during the nursing period. These findings are in contrast to our previous observations of increased adiposity in female offspring and decreased lean mass in male offspring when we used a different dosing paradigm and PCBs were given prior to and during pregnancy and during nursing (30). Recent work from our research group demonstrates consistent and pronounced alterations in offspring later-life body composition as a result of exposure to PCBs solely during gestation, where 4-month-old adult offspring exposed to PCB126 exhibited significantly reduced lean mass profiles when compared to vehicle-exposed offspring (32). Unlike the effects of gestational PCB exposure on offspring lean mass profiles, offspring fat mass profiles respective to PCB exposure were modulated by the presence or absence of nuclear factor erythroid-2-related factor 2 (Nrf2) (32), a key transcription factor responsible for regulating the detoxification of coplanar PCBs. Those adult offspring born to Nrf2 knockout dams exposed to PCBs during pregnancy exhibited significantly increased adiposity when compared to vehicle control offspring (32). Taken together, this suggests that *in utero* PCB exposure, or a combination of timed exposures during development, is more detrimental than nursing exposure alone as it pertains to obesity risk, or body composition changes, associated with early-life PCB exposure.

Iterations of *in vivo* PCB-induced glucose impairments have been extended to *in vitro* situations, as PCB exposure has been shown to inhibit insulin-stimulated glucose uptake in adipocytes (23, 42) and hepatocytes (42). Interestingly, in myotubes, PCB126 exposure does not affect insulin-stimulated glucose uptake, but has been demonstrated to reduce basal glucose uptake (26). In the present study, following glucose challenges, we observed impairments in the glucose disposal of offspring exposed to PCB126 during the nursing period compared to vehicle controls. Dams in this study were exposed to PCB126 or vehicle on PNDs 3, 10, and 17. It is important to note that the maternal dose at PND17 may not contribute substantial levels of PCBs to the offspring as they likely would consume little milk after this age. Our observation is largely consistent with the aforementioned *in vitro* studies as we demonstrate the ability of PCBs to alter glucose tolerance. Here, our observation of the PCB-induced glucose impairment in offspring was short-term. Specifically, both male and female offspring nursed to PCB-exposed dams had significantly elevated blood glucose levels at weaning (**Figure 3**). By twelve weeks of age, neither male nor female offspring presented any impairments in response to the PCB exposure (**Figure 3**). We previously demonstrated that PCB126 exposure prior to and during gestation (not during nursing) significantly impairs glucose disposal after a glucose challenge in animals (32). This suggests that the *in utero* period is the most susceptible window for long-lasting PCB-induced glucose intolerance because nursing only PCB exposure caused glucose tolerance that was reversible over time. In mice, persistent oral Aroclor 1254 exposure induced glucose intolerance and hyperinsulinemia while cessation of the exposure abolished PCB-induced detriments (45). Such data could explain the lack of permanence in the glucose impairments observed in the offspring of the current study, as PCB exposure through the dams’ milk serves as a persistent direct exposure for offspring from birth until weaning.

There are many positive outcomes of nursing for mothers and babies. Health benefits observed in adults that were breastfed include, but are not limited to, increased cognitive functioning, decreased obesity in childhood and adulthood, and reduced T2D risk (46, 47), thus, suggesting a beneficial developmental impact of breastfeeding on health span. Additionally, moms that breastfeed their infants have reduced rates of cancer, obesity, T2D, and heart disease after parturition when compared to those women who did not (46). While beneficial effects of nursing are apparent, adverse effects associated with toxicant exposure have also been observed (48). Despite potential risks that may occur *via* increased exposure to pollutants, health care providers encourage mothers to breastfeed as the benefits outweigh the risks.

Although we found that short-term early-life impairments in offspring glucose tolerance results from PCB126 exposure during the nursing period, more work is needed to understand how and why each period of perinatal PCB exposure collectively and independently influence diabetes risk in offspring. A limitation of our work is that we did not assess the PCB126 body burden after exposure in dams and offspring. Future studies will address this limitation by quantifying the amount of PCB126 accumulated in dam milk and offspring adipose tissue and serum. Exposing dams daily or on a weekly basis starting at PND0 could also affect the offspring metabolic outcomes differently. Additionally, the study could have benefited from more robust metabolic phenotyping and mechanistic assays, such as quantification of insulin resistance and adipocyte dysfunction to better understand observed offspring health outcomes. This is a limitation of the current work, and these experiments could be included in future studies. Regardless, we feel the body weight, body composition, and glucose tolerance data collected following nursing only exposure to PCB126 contribute important information to the field.

In conclusion, this study demonstrates that glucose impairments that occurred in offspring exposed to PCB126 during nursing were short-term. Thus, when compared to our previous work (30, 32) these results suggest that *in utero* exposure potentially poses more detrimental health effects on offspring when compared to nursing exposure. The short-term early life impairments are transient and disappear as offspring age. This suggests a direct toxicity effect, but it is uncertain whether there will be residual effects much later in life. While toxicological exposures during the nursing period may pose a threat to long-term health outcomes of offspring, the benefits of breastfeeding outweigh the risks. Although toxic exposures during nursing have been shown to be less harmful than *in utero* exposure, our work demonstrates they should not be ignored given that nursing and *in utero* exposure often co-exist. Therefore, knowledge of the physiological interaction between the two is warranted.

## Data availability statement

The raw data supporting the conclusions of this article will be made available by the authors, without undue reservation.

## Ethics statement

The animal study was reviewed and approved by University of Kentucky Institutional Animal Care and Use Committee.

## Author contributions

BR and KS both made substantial contributions and share equal first authorship. SN, HS, and KP contributed to the conception and design of this study. KS, SN, and MW contributed to data acquisition. BR, KS, and SN contributed to data analysis. BR, KS, SN, LR, CR, HS, and KP contributed to the interpretation of the data. BR, KS, SN, MW, LR, CR, HS, and KP drafted and critically revised the manuscript, gave final approval, and agree to be accountable for all aspects of work ensuring integrity and accuracy. All authors contributed to the article and approved the submitted version.
